# Are body circumferences able to predict strength, muscle mass and bone characteristics in obesity? A preliminary study in women

**DOI:** 10.7150/ijms.41713

**Published:** 2020-03-15

**Authors:** Valentina Cavedon, Chiara Milanese, Carlo Zancanaro

**Affiliations:** Laboratory of Anthropometry and Body Composition, Department of Neurosciences, Biomedicine and Movement Sciences, University of Verona, Verona, Italy

**Keywords:** anthropometry, body composition, mineral mass, mineral density, linear regression, prediction equation

## Abstract

Measurement of body circumferences (BCs) is widely used as an anthropometric tool to assess body composition and health risk in obese individuals. In this preliminary work we evaluated the association of several BCs with Dual-energy X-ray Absorptiometry (DXA)-measured lean mass as well as leg press test scores with an aim at exploring the potential of BCs as predictor of body composition and muscle strength. A total of 34 female participants aged 47.3±7.6 y who were obese (BMI, 30.4-43.7 kg/m^2^) were recruited. The upper arm (relaxed), wrist, chest, waist, hip, thigh, and calf circumferences were measured. The skinfold-corrected muscle (including bone) circumferences at the arm, thigh, and calf site were also calculated. Lean mass components were measured by DXA with a Hologic QDR Explorer scanner according to the manufacturer's procedures. Lower limbs strength was assessed with the 1-Repetition Maximum leg press. Bivariate association between variables was assessed with the Spearman's correlation coefficient after the Benjamini and Hochberg False Discovery Rate procedure. Predictive equations were developed using stepwise multiple regression analysis. Several statistically significant correlations (Benjamini and Hochberg corrected P [P_c_] < 0.05) were present between BCs and DXA-measured body composition variables, and leg press test scores with special regard to the chest, arm, waist, and hip circumferences. Multiple regression analysis yielded statistically significant predictive models (P_c_ < 0.05 for all; adjusted R^2^ ranging 0.123 - 0.504; standard error of the estimate ranging 4.0% - 11% of the mean measured value) for all body composition as well as leg press outcomes. The current findings show that BCs represent a simple, suitable anthropometric measurement with a potential to predict several lean mass components as well as lower limbs strength in obese females. The proposed predictors need to be validated in a larger sample of participants and in obese males.

## Introduction

The large majority of lean body mass i.e., the metabolically active component of the body is represented by skeletal muscle playing a key role in a number of physiologic processes 1. In turn, the large majority of skeletal muscle is appendicular (about 75% of total) with the lower limbs containing about 55% of the body skeletal muscle 2,3. When assessing skeletal muscle, both the absolute amount of muscle tissue (i.e., muscle mass) and the ability of muscle to generate strength (i.e., muscle quality) should be considered, because skeletal muscle mass and strength may not parallel in individuals 4.

In obesity, reliable assessment of skeletal muscle mass and strength would be relevant for both patients and physicians. For example, skeletal muscle mass is a major responsible for glucose uptake under insulin-stimulated conditions, thereby strongly affecting insulin sensitivity, which is frequently altered in obesity. Further, appendicular muscle mass is associated with mobility and functional independence, which may be a factor affecting the quality of life of obese people 5. On the other hand, muscle strength is an important component of physical function 6 and may affect habitual physical activity 7. Overall, assessing muscle mass and strength in obese persons could help evaluating their general physical conditions as well as providing an estimate of healthy and active lifestyle. Moreover, results of strength assessment could be useful for the monitoring of exercise programs for obese patients.

Traditionally, obesity is thought to be beneficial to bone in terms of bone mineral density (BMD) and/or content (BMC) via increased mechanical loading associated with higher fat mass and/or the extra strain imposed on bone by increased muscle mass 8; however, it has been suggested that locomotion-induced ground reaction forces and accompanying muscle contractions are sufficient to improve bone strength 9. Accordingly, the relationships between body mass, body composition and bone mass/quality are complex 10 and the impact of obesity on bone status is not completely clarified, the effects of body fat and lean mass on bone being possibly different 11. However, independently of the actual mechanism(s) mediating the relationship between body composition and bone, estimation of bone characteristics in obesity would be a useful complement to the routine evaluation of patients with special reference to females, which are at increased risk of osteoporosis.

**Figure 1:**
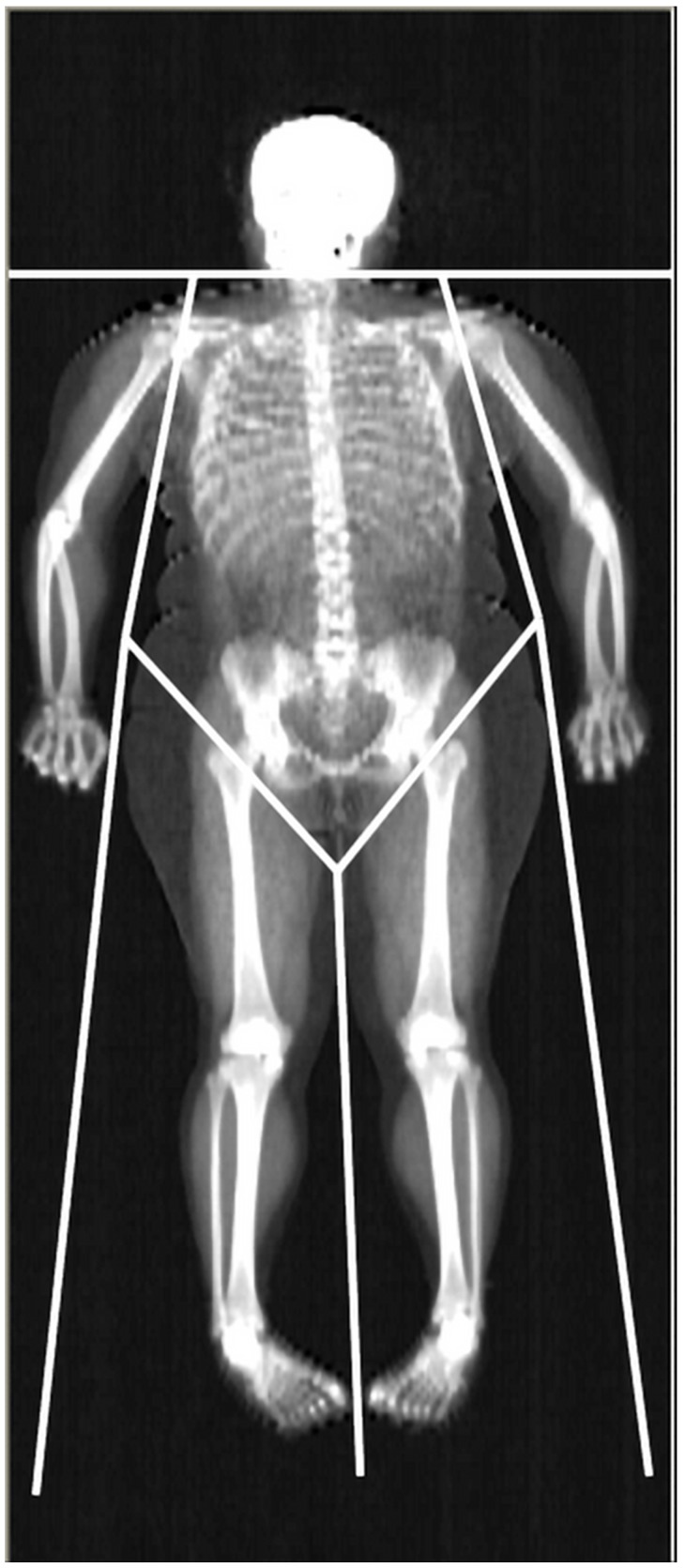
Representative whole-body DXA scan. The limits defining the regions of interest used in this work (upper and lower limb, trunk) are depicted.

Dual-energy X-ray Absorptiometry (DXA) is able to accurately assess lean mass (distinguishing between the mass of lean soft tissue, LST, and mineral mass) at the whole body (WB) and regional level. The areal BMD is also included in the standard DXA output. The limb LST mass, BMC, and BMD can be easily obtained from DXA readings (Fig. 1). The sum of LST mass in the four limbs i.e., appendicular LST mass is used as a surrogate of skeletal muscle mass, and DXA estimates of appendicular LST mass have been validated against skeletal muscle mass measurements obtained with magnetic resonance imaging and computed tomography 3, 12, 13. As far as muscle strength is concerned, isotonic muscle strength can be measured using several techniques, one of the most commonly used being the One Repetition Maximum (1-RM) technique. The Repetition Maximum represents the maximum number of repetitions performed before fatigue prohibits completion of an additional repetition and generally reflects the intensity of the exercise 14. However, both DXA and 1-RM are laboratory-based techniques, which are unpractical for use in large or field studies due to their relatively high costs and logistics required to perform measurements. Further, DXA employs X-ray radiation, which may hamper its widespread use, and 1-RM may be harmful to joint and muscle when measuring maximum muscle strength in persons with low muscular strength. According to the considerations above, there is a need for a non-invasive, cheap, and repeatable tool to estimate lean mass components and muscle strength in obesity.

Body circumferences (BCs) are simple to measure, inexpensive and non-invasive, and require minimum instrumentation (just an anthropometry tape) while yielding accurate data when performed by a trained operator according to standard procedures 15. Moreover, BCs can easily be used in field studies as well as the clinical setting. In addition to the ease of measurement, BCs have fewer problems with measurement error vs. skinfold thickness, especially in persons with obesity 16.

Several BCs have been used to estimate WB muscle mass as well as muscle strength especially the mid-arm, mid-thigh, and maximum calf circumference 15, 17-21. Anthropometric predictive equations have been developed for appendicular muscle mass 21-26. The association between bone mineral mass and density, and anthropometry has been explored as well leading to derivation of predictive anthropometric equations for bone 27-29. More recently, it has been shown that calf circumference is able to predict BMD in older adults 30, arm circumference at muscle flexion is predictive of lumbar spine BMD 31 and waist circumference is inversely associated with BMD in male and female Koreans >50y after adjusting for several confounders 32. However, the predictive equations above were generated in the general population or in populations with special characteristics and may be not accurate in obese patients. In summary, validating BCs as estimators of muscle mass and strength as well as bone characteristics in obesity would be of use to physicians, researchers, and patients.

In this preliminary work, a group of obese females underwent BC measurements, DXA body composition analysis, and lower limbs strength tests with an aim at exploring the following hypotheses: 1. BCs are associated with LST mass of the limbs (a proxy of appendicular skeletal muscle) and muscle strength as well bone mineral characteristics, and 2. BCs are suitable predictors of lean mass variables and muscle strength.

## Materials and Methods

### Participants

Thirty-four Caucasian adult female obese (body mass index, BMI >30kg/m^2^) outpatients (age range 35-61y; 38% postmenopausal) previously submitted to lifestyle counselling and with body weight stable over the last three months participated in this study. No patients suffered from diabetes or other significant disease nor had history of osteoporosis; none of them had been taking medications in the last six months that could potentially interfere with the evaluations carried out in the study. This study was carried out in accordance with the recommendations of the Helsinki Declaration with written informed consent obtained from all participants. The study protocol was approved by the Institutional Review Board at the University of Verona.

### Anthropometry

Body mass and stature were taken at the nearest 0.1 kg and 0.01 m with a Tanita electronic scale BWB-800 MA (Wunder SA.BI. Srl) and a stadiometer (Holtain Ltd., Crymych, Pembs. UK), respectively. Body circumferences were measured at the upper arm (relaxed), wrist, chest, waist, hip, thigh and calf site according to standard procedures 33. Since skinfold-corrected limb circumferences may provide a measure of corresponding appendicular lean limb circumference 17, 19, 20, the arm, thigh, and calf circumferences were corrected for subcutaneous adipose tissue thickness. Skinfold thickness was taken at the triceps, anterior thigh, and calf site according to standard procedures 34. The corrected muscle (including bone) circumferences were calculated as Cm = C_limb_ - πS/10, where Cm = corrected muscle circumference, C_limb_ = limb circumference (cm), and S = skinfold thickness (mm), assumed to be twice the subcutaneous adipose tissue thickness. The percent technical error of measurement was <1.0% for BCs and <3.5% for skinfolds recorded in this study and therefore within acceptable limits (i.e., <5.0% for skinfold thickness and <1.0% for all other measures) 34.

### Body composition analysis

Body composition was evaluated by means of DXA (QDR Explorer W, Hologic, MA, USA; fan-bean technology, software for Windows XP version 12.6.1) according to the manufacturer's procedures. The scanner was quality-checked daily against the standard supplied by the manufacturer to avoid possible baseline drift. In our laboratory, the precision error (percent coefficient of variation with repositioning) of whole body (WB) DXA measurements is 1.1%, 0.9%, 2.3%, 2.8% and 0.5% for BMC, BMD, fat mass (FM), percentage fat mass (%FM) and LST mass, respectively. The same operator performed all scanning and analysis to ensure consistency.

For the standard regional body composition estimations, Hologic software readings divided the body into trunk, entire upper limb, entire lower limb, and head (Fig. 1).

BMC is preferable to BMD as bone health outcome. Actually, BMD is more prone to measurement error because DXA measures areal, not volumetric, BMD. Accordingly, BMD may artificially over- or underestimate the relative bone mass according to the individual's bone size 35. Moreover, systematic inaccuracies in BMD measurement with DXA may ensue 36. Therefore, BMC was considered the main index of bone quality in this work; however, in order to allow comparison with most of the previous relevant literature, BMD data are also presented and discussed. The WB less head (WBLH) DXA measurements for BMC and BMD were used as dependent variable in regression analysis (vide infra) instead of WB values with an aim at increasing precision. In fact, the skull has a relatively large impact on the WB BMC and BMD due to its large content in compact bone; however, the skull is not involved in weight bearing and correlation of head BMD with that of other parts of the skeleton is far from perfect 37; moreover, changes in bone density of the head with age and BMI differ to that of the rest of the skeleton 38. WBLH measurements were taken by excluding the head from the rest of the body by a plane at 90° to the long axis of the body, immediately caudally to the mandible. In this study, LST mass and FM as well as BMC and BMD values, and %FM were also calculated for the right and left upper limbs combined (region: upper limbs), the right and left lower limbs combined (region: lower limbs), and the upper and lower limbs combined (region: appendicular).

### Strength test

The maximum dynamic strength of lower limbs was determined on leg press test (Personal Selection TÜV, Technogym, Italy). The 1-RM method was used. The initial load for the 1-RM test was predicted from 50% of body weight. After a warm-up of 5 minutes walking at self-selected speeds on a treadmill followed by one set of 10 repetitions at a relatively light load, each participant performed a single repetition with a weight she could lift through a complete range of motion. At the conclusion of each successful lift, a higher load (2.5-5.0%) for a second trial was added. A recovery period of one minute was allowed between lifts. This procedure was repeated until the participant could no longer lift the weight (achieved in 3 to 6 attempts). The greatest amount of weight lifted successfully was recorded as the 1-RM, and the test's score was determined with the formula [lifted load/1.0278 - (0.0278 x numbers of repetition)] 39. Strength relative to whole body mass and LST mass was calculated as the test's score (kg) divided by whole body mass (kg) and by DXA-measured LST mass (kg), respectively.

### Statistical analysis

Normality of data was checked with the Shapiro-Wilks test. Data were summarized as mean ± standard deviation and median (interquartile range) for normally and non-normally distributed variables, respectively. Bivariate and partial association between variables was assessed with the Spearman's correlation coefficient, ρ. The strength of the correlation coefficient was considered small (0 - 0.30), moderate (0.31 - 0.49), large (0.50 - 0.69), very large (0.70 - 0.89), and almost perfect (0.90 - 1) as per Hopkins 40. The Benjamini and Hochberg False Discovery Rate procedure was used to get corrected p-value (p_c_), in order to minimize Type I error associated with multiplicity of correlations in the same dataset 41. False Discovery Rate was set at 0.05.

Stepwise regression analyses were conducted with DXA-measured outcomes or strength tests scores as the dependent variable and a maximum of three body circumferences showing the highest significant (P_c_ < 0.05) correlation with the dependent variable as the independent variables. The number of predictors in regression analysis was chosen to have a sample size/predictor variable ratio of about ten, which is currently used in regression analysis. The probability of F-to-enter was set at ≤ 0.05 for inclusion and ≥ 0.10 for exclusion of predictor variables. Adjusted R^2^ and SEE were used to assess the goodness of the predictor model.

Homoscedasticity of data was assessed by plotting the residuals of multiple regression analysis against the predicted values as well as the Koenker test 42. The presence of serial correlations among the residuals was tested using the Durbin-Watson statistic and the variance inflation factor (VIF) was calculated to check for multicollinearity in the multiple linear regression models. Statistical significance was set at P ≤ 0.05.

## Results

### Characteristics of participants

The demographic characteristics of the participants are summarized in Table 1. Out of 34 participants, 13 were Class I obese (BMI 30-34.9 kg/m^2^), 11 were Class II (BMI 35 - 39.9 kg/m^2^), and 10 were Class III (BMI ≥ 40 kg/m^2^) 43. Median age, stature, and body mass were 47.5 y, 1.57 m, and 87.85 kg, respectively. No participant was found to be sarcopenic according to the skeletal muscle mass (SMM) index 22. The SMM index is calculated as the ratio of DXA-measured appendicular LST (kg) and height (m)^2^; the cut-off value for sarcopenia (SMM < 5.45) corresponds to a SMM index less than two standard deviation below the mean of the reference population i.e., a young female group from the Rosetta study aged 18 to 40 22. In our sample of obese females, the mean value of the SMM index was 7.88 ± 0.92kg/m^2^. The DXA-derived T-score was above the current cut-off for osteoporosis (-2.5) in all participants.

### Body composition analysis and strength test outcomes

The results of DXA measurements are reported in Table 2.

The results of leg press test are presented in Table 3. When the sample of obese females was subdivided according to median age (<48y, n = 18; > 48y, n = 16), average scores in strength tests were lower in older participants, but the difference was not statistically significant (202.3 ± 51.0 kg vs. 179.1 ± 33.1 kg, P = 0.130).

### Correlation between BCs, body composition and leg press test

**Figure 2:**
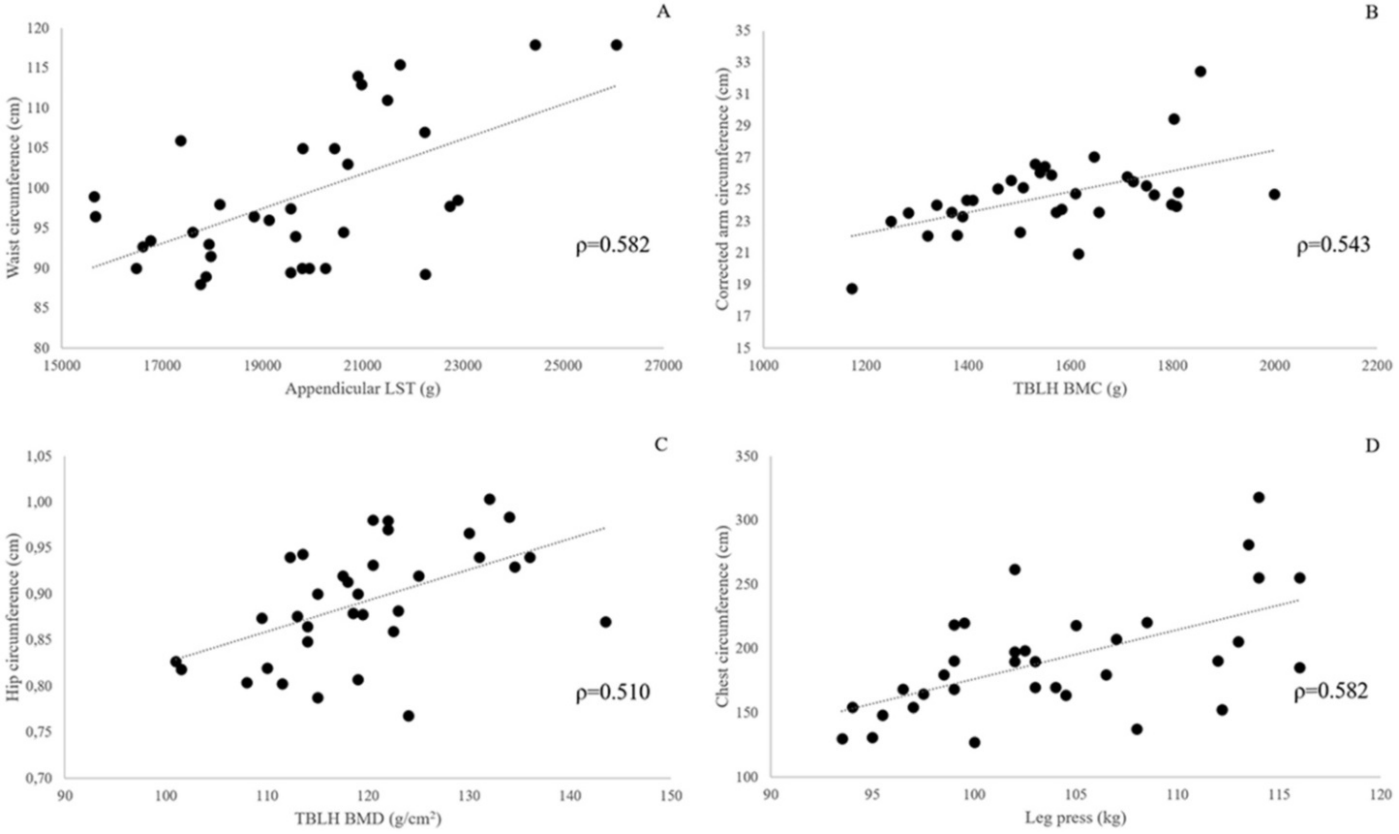
Panels A-D: scatterplots showing the association between selected body composition and strength test outcomes, and body circumferences in 34 obese females. All correlations are statistically significant at Benjamini-Hochberg corrected P-value <0.01.

After Benjamini & Hochberg correction, P values ≤ 0.025 were considered significant (P_c_ ≤ 0.05). Bivariate correlations between body composition and muscle strength variables, and BCs are presented in Table 4. Several statistically significant (P_c_ < 0.05) correlations were found between BCs and DXA-measured body composition variables and lower limbs strength tests scores. The larger number of significant correlations was found for the chest circumference (12 out of 15 body composition variables and the strength test scores), the arm and waist circumference (10/15), and the hip circumference (7/15). The pattern of association between selected measurement outcomes and BCs is graphically depicted in Fig. 2. Relative strength expressed either as leg press score/whole body mass or leg press score/LST mass did not show any significant correlation with BCs. Similar findings were obtained after adjusting for age.

Trunk circumferences (chest, waist, hip) showed stronger correlation with limb LST masses than regional with the exception of wrist circumference showing the strongest correlation with upper limbs LST. Upper and lower limbs LST did not significantly correlate with the corrected arm and corrected calf circumference, respectively.

WBLH BMC showed positive, large-to-moderate correlation with the chest, arm, and corrected arm circumference. Appendicular BMC showed the strongest correlation with the corrected arm circumference. Upper limbs BMC moderately correlated with the wrist circumference. Lower limbs BMC did not significantly correlate with regional circumferences. All BMD variables showed their strongest positive correlation with one of the trunk circumferences. No significant correlation was found between lower limbs BMD and the regional circumferences. Leg press scores showed large correlation with the chest and waist circumferences but were not significantly correlated with regional circumferences.

Most correlations between BCs and the measured outcomes were still significant after controlling for participants' age. In several instances, the strength of correlation increased after adjusting for age or correlation become statistically significant (Table 4).

### Regression analysis

The results of stepwise linear regression analyses are reported in Table 5. A statistically significant model was developed for all dependent variables using one or two BCs. Models explained about 30 to 50% of variance for TBLH and regional, 12.3 - 27.3% of variance for BMC variables, 33.3 - 42.8% of variance for TBLH, appendicular, and upper and lower limbs BMD, and 43.1% of variance for leg press. Adding age to the equation did not increase R^2^ in any of the previous models. Similarly, adding WBLH FM to the equation did not significantly increase R^2^ in any of the previous models, with the exception of leg press (+8.7%, P = 0.016) the resulting equation being: leg press = -550.4 + (5.614 x chest) + (6.132 x corrected thigh) + (-0.003 x WBLH FM). When WBLH LST was used as the predictor, the explained variance for leg press was 31.6% (P < 0.001; SEE = 36.8 kg): using lower limbs LST as the predictor variable, the explained variance was 37.1% (P < 0.001; SEE = 36.3 kg). In the equations reported in Table 5, the SEE for body composition variables and leg press test scores was 5 - 13% and about 16% of the mean measured value, respectively. For all models, the Durbin- Watson statistics was between 1.5 and 2.5, indicating that autocorrelation was not present in the residuals; VIF values for predictor variables were < 4 and the Koenker test was > 0.05, indicating that models are robust to collinearity and heteroscedasticity.

## Discussion

To the best of our knowledge, this is the first study investigating the relationships between BCs, DXA-measured lean mass components, and muscle strength in obese females. Results indicate that:

- Several statistically significant correlations are present between BCs and LST mass, BMC and BMD as well as performance in the leg press test.
- Body circumference has better potential in predicting muscle mass and strength than bone characteristics.

Our first hypothesis explored in this study was that relationships exist in obesity between BCs and lean mass components, and muscle strength. Results confirmed such a hypothesis by showing, in a sample of women encompassing a similar number of Class I, Class II, and Class III obese participants, several positive statistically significant correlations between DXA-measured lean mass variables and strength tests scores, and several BCs (Table 4). This indicates that in obese females increasing BCs generally reflect increasing amounts of lean soft mass and mineral mass, and higher muscle strength. Such an association was essentially independent of age, suggesting that it is inherent to the obese condition. A second hypothesis tested in this work was that BCs are able to predict lean mass variables and muscle strength. Results partially confirmed such hypothesis by showing that BCs are better predictors of muscle- than bone-related variables. Overall, findings indicate that BCs have potential as reliable predictors of selected lean mass components and strength test performance in obesity and prompt for further studies in a larger number of obese individuals of both sexes.

In this study appendicular LST mass, a proxy of body skeletal muscle 3, 12, 13, was measured with an accurate method such as DXA 44. Correlation analysis (Table 4) showed that in our sample of obese females, trunk circumferences are more representative of bodily skeletal muscle than limbs circumferences even after skinfold correction, despite trunk circumferences perimetering a substantial amount of visceral organ and fat tissue in addition to LST. This is in agreement with previous findings in heterogeneous populations showing that limb anthropometry may be inaccurate in estimating muscle cross-sectional area with increasing limb adiposity 46-48. In the obese females participating in this study, DXA-measured trunk mass was about 50% of body mass (Table 2), these two variables showing almost perfect correlation (ρ = 0.91).

Accordingly, it seems that the bodily skeletal muscle complement of obese females is more proportional to body mass than regional mass, independently of loading. Our results are supported by data 48 obtained in 110 middle age Korean diabetic women (age range 40-60 y; BMI 27.2 ± 2.7 kg/m^2^) showing small to moderate (albeit statistically significant) correlation between arm and thigh circumference, and DXA-measured TB LST mass (r = 0.368, P < 0.001; r = 0.226; P = 0.025). Unfortunately, the authors did not present results of correlation analysis between LST variables and another measurement taken in their work namely, the waist circumference thereby preventing more extensive comparison of theirs 48 and the current results. In the present study the wrist circumference more strongly correlated with upper arms LST (ρ = 0.692; P_c_ < 0.001) than any other circumference and the wrist circumference was the only circumference showing significant correlation with upper limbs BMC, albeit at the limit of statistical significance. The wrist circumference is traditionally used to calculate body-frame size 49 because it is relatively devoid of subcutaneous fat and skeletal muscle, but its relationship with regional skeletal muscle or mineral mass is not established. Since wrist circumference has been considered a possible risk factor for developing diabetes, metabolic syndrome and cardiovascular disease 50, 51 the relationships between wrist circumference and skeletal and mineral muscle mass in obesity deserve further investigation.

BCs were able to predict limb LST mass, explaining about 32 to 50% of in-sample variance (adjusted R^2^, 0.318 - 0.504; Table 5). The SEE for appendicular LST mass was about 2.0 kg i.e., 10.1 % of the mean DXA-measured value (Table 2). This result compares well with that obtained in larger groups of young adult non-obese males and females 52-54. Estimation of appendicular LST mass in obesity is of interest because it is significantly associated with insulin levels in overweight or obese women, independently of age, body size or fat mass 55 and increasing skeletal muscle is associated with decreased insulin sensitivity 56. Accordingly, the proposed predictive equation could be of use in the non-invasive evaluation of skeletal muscle during weight change.

Correlation analysis (Table 4) showed that upper body circumferences are more representative of the local and bodily amounts of bone mineral in obese females than that of the lower body, suggesting that the association between BMC and BCs is independent of loading. At variance, lower limb circumferences showed stronger average correlation with regional and WBLH DXA-measured FM and %FM than upper limb circumferences (not shown). Therefore, it can be hypothesized that higher BMC in obese females is largely independent of regional and body FM. Upper arm circumferences were prevalently selected as predictors of BMC in regression analysis (Table 5) in accordance to the higher strength found in correlation analysis (Table 4). However, the ability of BCs to predict BMC was limited (adjusted R^2^ ranging from 0.123 to 0.273), the SEE for WBLH BMC being about 11% of the mean DXA-measured value indicating that the relevant anthropometric predictive equation should be used with caution in individual. In our sample of obese females, the chest and/or hip circumference showed strongest correlation with BMD variables (Table 4) indicating that body tissue perimetered in the trunk is more representative of WBLH and regional BMD than that in the limbs. The hip circumference was the only common predictor for all BMD outcomes in regression analysis, the chest circumference being selected as an additional predictor in two out of four equations (Table 5).

These findings are supported by previous data showing that hip circumference was more strongly associated with BMD at both the femoral neck and lumbar spine site than BMI, waist circumference, body FM and appendicular FM in a large number of obese males and females 57. The SEE for WBLH BMD was about 5% of the mean DXA-measured value, which is reasonable accuracy. The explained variance was generally higher than that obtained for BMD at several key body sites using age and anthropometry in adult females 27. Accordingly, the anthropometric predictive equations presented herein would be of some use in estimating the BMD of obese females.

The relationship between obesity and muscle strength is controversial. It has been shown that muscle strength is closely related to the absolute amount of skeletal muscle 58, 59 the latter being frequently increased in obese persons, and it has been hypothesized that weight bearing and supporting of the larger body mass have a training effect in the obese 60. Other works showed a negative impact on skeletal muscle from adolescence 61 to old adulthood 24. Findings on the relationship between muscle strength and anthropometric measurements such as BMI and body fat percentage were also inconclusive. Work investigating the effect of obesity on muscle strength over a wide range of ages showed that absolute strength is increased in obese vs. non-obese individuals, strength being lower in the lower limbs musculature when normalized to total body mass (review in 62). In the current work, the strength of the correlation between thigh circumference and leg press was about half of that with the chest and waist circumference (Table 4). A similar correlation between thigh circumference and 1-RM leg press (r = 0.276) was found in a previous work carried out in obese females 48. The correlation between corrected thigh, calf and corrected calf circumference, and leg press scores was even lower (ρ ranging from 0.131 to 0.296). Overall, these data strongly suggest that lower limb circumferences are not very representative of lower limbs muscle strength in obese females. Accordingly, in obese females a weak association (r, 0.29 - 0.49) has been found between lower limbs strength and fat-free mass 63-65. Further support to this suggestion comes from the similarity between the strength of correlation between upper limbs and trunk BCs, and leg press scores presented in the current work (Table 4) and that found between maximal voluntary torque of the lower limbs, and body and lower limbs fat free mass (r = 0.46 and r = 0.57, respectively) in a larger sample (n = 132) of obese male and female patients (mean age = 40.5 ± 9.79 y, mean body mass = 126.13 ± 19.64 kg) 66. In our sample, no statistically significant correlation was found between BCs and relative muscle strength (leg press score/whole body mass or leg press score/LST mass) even after adjusting for age, showing that BCs are not suitable for predicting relative muscle strength in obesity. While lower relative muscle strength has been consistently found in obese vs. non-obese persons (review in 62), little attention has been given to differences in relative muscle strength within the obese population, apart when sarcopenic obesity is present. In this work involving non-sarcopenic participants spanning a wide range body weights, the lack of correlation between BCs and relative muscle strength even after adjusting for age suggests that lower limbs muscles were able to generate similar strength per unit body mass or unit lean mass in obese women irrespective of age. This prompts for more detailed investigation of skeletal muscle function in the obese population.

BCs revealed able to predict leg press scores with some accuracy (adjusted R^2^ = 0.431; SEE = 4.0% of the mean DXA-measured value). In normal weight females 67 anthropometry was able to predict bench press scores with a maximum R^2^ = 0.410 and SEE = 5.4 kg (18.9% of mean bench press score). In 39 premenopausal women aged 36 ± 8 y 69 a moderate, significant correlation (r) was found between plantar flexor maximum voluntary contraction and calf circumference (r = 0.584) and estimated muscle + bone cross-sectional area (r = 0.447). In linear regression analysis, R^2^ was 0.341 and 0.199 when the predictor was the calf circumference and the estimated muscle + bone cross-sectional area, respectively (P < 0.05 for both; SEE not available). Comparing the results of the present study with literature data, it is concluded that anthropometry is better able to predict muscle strength in obese than normal weight females. In our sample, BCs predicted leg press scores better than WBLH LST and lower limbs LST (explained variance = 43.1% and SEE 33.54 kg; 31.6% and 36.8 kg; 37.1% and 36.3 kg, respectively).

In agreement with the maintenance of most of the statistically significant correlations between BCs, and body composition and strength test scores after adjusting for age (Table 4), adding age to the developed predictive equations presented in Table 5 did not significantly change R^2^, indicating that equations are suitable for use across several decades of obese women chronological age.

In this preliminary work sample size was limited and the developed predictive equations were not validated in an independent sample. This obviously limits the generalized use of equations in the obese female population; moreover, we were not able to control for habitual physical activity in participants. However, the number of obese Class I, Class II, and Class III participants was roughly similar in the sample and the means, standard deviations, and ranges shown in Table 1 for age, weight and anthropometric measures indicate that participants were heterogeneous enough for the development of non-population specific regression equations. As participants spanned a relatively large age range (35-61 years) and aging is associated with a decrease in maximal muscle force 69, this may have confounded any effect of obesity on skeletal muscle force. However, no participant was sarcopenic (SMM = 6.26 or higher) and no significant difference between participants aged below and above the sample median age (48y) was found in the outcome of leg press test, suggesting that age had a limited effect on results.

## Conclusion

In conclusion, this work demonstrated in obese females that increasing BCs positively correlate with skeletal muscle mass and strength as well as mineral bone, and BCs are suitable to estimate several such variables. Accordingly, BCs represent a promising tool for estimating skeletal muscle, muscle strength and bone health variables when direct measurements are not available/feasible. Further work in larger samples of obese females and in obese males is needed to generalize the proposed predictive anthropometric in the obese population.

## Figures and Tables

**Table 1:** Demographic characteristics of the 34 obese females participating in this study.

Variable	Mean ± SD / Median (IQ)	Maximum/Minimum
**Age (y)**	47.3 ± 7.6	35 - 61
**Stature (cm)**	158.2 ± 4.3	151 - 170
**Body mass (kg)**	90.9 ± 10.6	72.9 - 109.3
**BMI (kg/m^2^)**	36.1 (8.7)	30.4 - 43.7

Normally distributed variables are presented as mean ± standard deviation, SD); non-normally distributed variables are median (interquartile range, IQ). BMI, Body Mass Index.

**Table 2:** Body circumferences and DXA-measured body composition of study participants.

Variable	Mean ± SD /Median (IQ)	Maximum/Minimum
**Body circumference (C)**		
Mid-arm C (cm)	35.1 ± 2.7	30.0 - 43.4
Corrected mid-arm C (cm)	29.2 ± 2.5	24.4 - 36.4
Wrist C (cm)	16.7 ± 1.2	14.5 - 19.2
Chest C (cm)	103.9 ± 6.8	93.5 - 116.0
Waist C (cm)	96.5 (14.1)	88.0 - 118.1
Hip C (cm)	119.7 ± 9.6	101.2 - 143.5
Waist-to Hip ratio	0.83 ± 0.06	0.69 - 1.01
Mid-thigh C (cm)	63.0 ± 5.9	51.4 - 79.7
Corrected mid-thigh C (cm)	47.5 ± 5.1	35.4 - 63.1
Calf C (cm)	40.5 ± 3.3	33.2 - 49.5
Corrected calf C (cm)	30.6 ± 1.7	27.3 - 34.7
**DXA measurement**		
WBLH BMC (g)	1563.3 ± 196.1	1173.6 - 1998.7
WBLH BMD (g/cm^2^)	0.892 ± 0.062	0.768 - 1.003
WBLH LST (g)	44680.7 ± 5210.4	36661.6 - 55656.2
WBLH FM (g)	38737.7 ± 6544.0	27330.0 - 50718.5
WBLH FM (%)	45.3 ± 4.2	37.6 - 52.8
Upper limbs BMC (g)	289.4 ± 36.2	207.8 - 353.1
Upper limbs BMD (g/cm^2^)	0.717 ± 0.043	0.621 - 0.81.4
Upper limbs LST (g)	4417.5 ± 739.8	3500.4 - 6404-3
Upper limbs FM (g)	5314.2 ± 1165.1	3220.3 - 7486.6
Upper limbs FM (%)	52.1 ± 5.9	40.0 - 63.6
Lower limbs BMC (g)	721.9 ± 100.9	538.2 - 979.1
Lower limbs BMD (g/cm^2^)	1.062 ± 0.086	0.903 - 1.234
Lower limbs LST (g)	15182.8 ± 2005.7	11898.8 - 19649.3
Lower limbs FM (g)	13179.0 ± 3930.2	6508.9 - 22446.7
Lower limbs FM (%)	44.4 ± 6.8	28.7 - 57.3
Appendicular BMC (g)	1011.3 ± 125.1	766.5 - 559.3
Appendicular BMD (g/cm^2^)	0.933 ± 0.075	0.807 - 1.045
Appendicular LST (g)	19700.2 ± 2439.6	15629.7 - 26053.6
Appendicular FM (g)	18493.2 ± 4148.2	11016.5 - 28341-5
Appendicular FM (%)	46.6 ± 5.6	33.8 - 58.0

Normally distributed variables are presented as mean ± standard deviation, SD); non-normally distributed variables are median (interquartile range, IQ). BMC, Bone Mineral Content; BMD, Bone Mineral Density; LST, Lean Soft Tissue; FM, Fat Mass; WBLH, Whole-Body Less Head.

**Table 3:** Results (mean ± standard deviation, SD) of leg press strength test in the study participants.

Variable	Mean ± SD	Minimum/Maximum
**Leg press (kg)**	191.4 ± 44.5	127.1 - 318.0

**Table 4:** Bivariate correlations (Spearman's ρ) between BCs, DXA-measured body composition, and muscle strength test scores in the study participants.

Variable	Body circumference (cm)									
	Arm	Corrected arm	Wrist	Thigh	Corrected thigh	Calf	Corrected calf	Chest	Waist	Hip
**Lean soft tissue (LST)**										
Upper limbs LST (g)	0.154	0.259	0.692***	-0.270	-0.212	-0.138	0.266	0.513*	0.515*	-0.188
Lower limbs LST (g)	0.449*	0.398*°	0.289	0.479*	0.502*	0.472*	0.340	0.451*	0.518*	0.562*
Appendicular LST (g)	0.416 *	0.406*°	0.448*°	0.312	0.348	0.346	0.360	0.526*	0.582**	0.405*
**Bone mineral content (BMC)**										
WBLH BMC (g)	0.462*	0.543*	0.319§	0.168	0.146	0.187	0.086	0.511*§	0.360	0.368
Upper limbs BMC (g)	0.193	0.376	0.387*§	-0.224	-0.189	-0.097	0.087	0.287	0.130	-0.237
Lower limbs BMC (g)	0.394*	0.444*	0.243	0.187	0.146	0.244	0.085	0.454*§	0.304	0.346
Appendicular BMC (g)	0.374	0.467*	0.308§	0.086	0.063	0.169	0.063	0.449*§	0.283	0.210
**Bone mineral density (BMD)**										
WBLH BMD (g/cm^2^)	0.468*	0.470*	0.150	0.250	0.175	0.216	-0.014	0.559*§	0.460*§	0.510*
Upper limbs BMD (g/cm^2^)	0.500*	0.412*°	0.316§	0.475*§	0.424*	0.355	-0.008	0.467*§	0.456*§	0.637***§
Lower limbs BMD (g/cm^2^)	0.409*	0.358	0.098§	0.226	0.116	0.188	-0.153	0.517*§	0.473*§	0.457*
Appendicular BMD (g/cm^2^)	0.480*	0.395*°	0.152	0.359	0.255	0.297	-0.100	0.449*§	0.506*§	0.604**
**Strength test**										
Leg press score (kg)	0.411*	0.453*°	0.154	0.285	0.296	0.169	0.131	0.582**§	0.491*§	0.312

*, P_c_<0.05; **, P_c_<0.01; ***, P_c_<0.001; P_c_, Benjamini-Hochberg corrected p-value; °, correlation no longer significant after adjusting for age; §, correlation improving/appearing after adjusting for age; WBLH, Whole-Body Less Head

**Table 5:** Results of stepwise linear regression analysis using DXA-measured body composition and lower limbs strength tests outcomes as the dependent variable and body circumferences as predictor variable(s).

Dependent variable	Predictor variable	Adjusted R^2^	Constant	B coefficient	SEE	P-value
**Lean soft tissue (LST)**						
Upper limbs LST	wrist	0.462	-2826.380	440.295	542.4	<0.001
Lower limbs LST	waist, corrected thigh	0.504	-5996.850	116.908, 202.234	1411.9	<0.001
Appendicular LST	waist	0.318	4283.673	155.779	2014.2	<0.001
**Bone mineral content (BMC)**						
WBLH BMC	corrected arm	0.273	448.618	45.304	167.2	0.001
Upper limbs BMC	wrist	0.123	88.295	12.060	33.9	0.024
Lower limbs BMC	chest	0.181	18.382	6.771	91.3	0.007
Appendicular BMC	corrected arm	0.194	339.661	24.858	112.3	0.005
**Bone mineral density (BMD)**						
WBLH BMD	chest, hip	0.413	0.151	0.004, 0.003	0.0485	<0.001
Upper limbs BMD	hip	0.387	0.368	0.637	0.0346	<0.001
Lower limbs BMD	chest, hip	0.333	0.155	0.005, 0.003	0.0691	0.001
Appendicular BMD	hip, waist	0.428	0.276	0.003 0.002	0.0514	<0.001
**Strength test**						
Leg press	chest, corrected thigh	0.431	-378.904	4.063, 3.117	33.54	<0.001

R^2^, coefficient of determination; SEE, standard error of the estimate.
